# The Homeodomain Protein Defective Proventriculus Is Essential for Male Accessory Gland Development to Enhance Fecundity in *Drosophila*

**DOI:** 10.1371/journal.pone.0032302

**Published:** 2012-03-12

**Authors:** Ryunosuke Minami, Miyuki Wakabayashi, Seiko Sugimori, Kiichiro Taniguchi, Akihiko Kokuryo, Takao Imano, Takashi Adachi-Yamada, Naoko Watanabe, Hideki Nakagoshi

**Affiliations:** 1 Graduate School of Natural Science and Technology, Okayama University, Okayama, Japan; 2 Department of Life Science, Faculty of Science, Gakushuin University, Tokyo, Japan; 3 Institute of Biomolecular Science, Gakushuin University, Tokyo, Japan; 4 Department of Biology, Graduate School of Science, Kobe University, Kobe, Japan; 5 Department of Biomolecular Science, Faculty of Science, Toho University, Funabashi, Japan; University College London, United Kingdom

## Abstract

The *Drosophila* male accessory gland has functions similar to those of the mammalian prostate gland and the seminal vesicle, and secretes accessory gland proteins into the seminal fluid. Each of the two lobes of the accessory gland is composed of two types of binucleate cell: about 1,000 main cells and 40 secondary cells. A well-known accessory gland protein, sex peptide, is secreted from the main cells and induces female postmating response to increase progeny production, whereas little is known about physiological significance of the secondary cells. The homeodomain transcriptional repressor Defective proventriculus (Dve) is strongly expressed in adult secondary cells, and its mutation resulted in loss of secondary cells, mononucleation of main cells, and reduced size of the accessory gland. *dve* mutant males had low fecundity despite the presence of sex peptide, and failed to induce the female postmating responses of increased egg laying and reduced sexual receptivity. RNAi-mediated *dve* knockdown males also had low fecundity with normally binucleate main cells. We provide the first evidence that secondary cells are crucial for male fecundity, and also that Dve activity is required for survival of the secondary cells. These findings provide new insights into a mechanism of fertility/fecundity.

## Introduction

In many higher insects, the reproductive behavior of females drastically changes after mating [1], [2]. Stimulation of egg laying and suppression of remating are induced by factors present in the male seminal fluid. The *Drosophila* male accessory gland secretes accessory gland proteins (Acps) into the seminal fluid, which are essential for male fertility/fecundity [3], [4]. Each of the two lobes of the accessory gland is composed of two types of binucleate cell: about 1,000 main cells and 40 secondary cells [5]. Adult main cells are flat hexagonal cells and secondary cells are large spherical cells interspersed among the main cells at the distal tip of each accessory gland lobe. A well-known Acp, sex peptide (SP, also known as Acp70A), is secreted from the main cells and induces long-term postmating response, such as increased egg laying and reduced sexual receptivity, to increase progeny production [6], [7], [8], [9], [10]. These postmating responses are critically regulated through SP binding to the G-protein-coupled SP receptor in the female reproductive tract [11], [12], [13]. In addition, SP-sperm interaction is also required for long-term postmating response through localization of SP to sperm storage organs, and the C-terminal part of SP is gradually released from sperm tails [14], [15]. In contrast to the increasing knowledge of Acps secreted from the main cells, little is known about physiological significance of the secondary cells.

Cell-fate determination of accessory gland primordia depends on fibroblast growth factor (FGF) signaling, and the mesodermal cells expressing an FGF receptor, Breathless (Btl), are recruited into a part of the male genital disc during late larval development [16]. The *btl*-expressing cells become epithelial, and give rise to accessory glands (paragonia) and seminal vesicles (vas deferens). Subsequent cell proliferation and functional differentiation of the accessory gland are regulated by the homeodomain transcription factor Paired (Prd) [17]. Mutant males for *prd* are sterile as they have severely reduced or no accessory glands [18], [19], [20], indicating that seminal fluid components from the accessory gland are essential for male fertility.

The homeodomain transcriptional repressor Defective proventriculus (Dve) is involved in various functions including wing morphogenesis, leg joint formation, head vertex specification, ommatidial cell-type specification, and functional specification of the midgut [21], [22], [23], [24], [25], [26], [27]. Here, we provide evidence that Dve is required for male accessory gland development, binucleation of main cells and survival of secondary cells, and also that secondary cells are essential for male fecundity.

## Results

### Spatio-temporal pattern of Dve expression during accessory gland development

**Figure 1:**
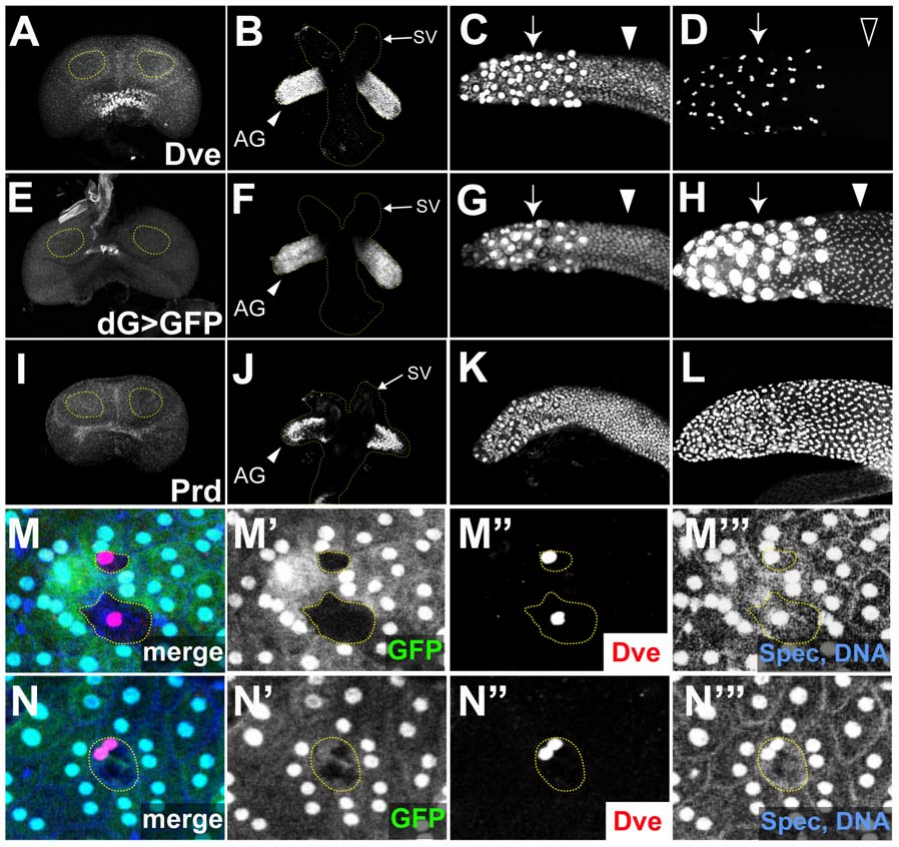
Dve expression during accessory gland development. (A–D) Dve protein expression. (E–H) *dve* enhancer activity (*w*; *UAS-GFP.nls8/+*; *dG35A/+*). (I–L) Prd protein expression. (A, E, and I) At the third-larval instar, male genital discs do not express Dve and Prd in accessory gland precursor cells (outlined). (B, F, and J) Accessory gland (AG) primordia at 24 hr APF start to express Dve (B) and Prd (J) (arrowheads). The shape of genital disc is outlined, and arrows indicate the seminal vesicle (SV). Accessory glands at 72 hr APF (C, G, and K) and of 1-day old adults (D, H, and L). Dve is expressed strongly in secondary cells (arrows in C, D) and weakly in pupal main cells (arrowhead in C). Dve expression in the adult main cells is undetectable (open arrowhead in D). The *dve* enhancer activity is nearly identical to the endogenous Dve protein expression (E–G), although the weak activity in main cells is detectable in the adult stage (arrowhead in H). Prd expression is detected in both types of cell from pupal to adult stages (K and L). (M and N) *prd* mutant clones of 4–6 days old adults are labeled with the absence of GFP expression (green), and are outlined with broken lines. Dve expression (red) is strongly derepressed in *prd* mutant main cells, which are mononucleate (M), whereas binucleation and Dve expression are unaffected in a *prd* mutant secondary cell (N). Nuclei (DNA) and cell membrane are labeled with TO-PRO3 and anti-Spectrin antibody, respectively (blue). Single channel images are shown in M′-M″′ and N′-N″′.

We have found that Dve is expressed in the male accessory gland at least from 24 hr after puparium formation (APF) but not in the male primordia of the genital disc in the late third-larval instar (Figure 1A, 1B). Dve is expressed strongly in secondary cells and weakly in main cells at 72 hr APF (Figure 1C), but Dve expression in main cells is undetectable in the adult stage (Figure 1D). To generate a GAL4 driver line that can induce gene expression during early stages of accessory gland development, we established a dve-GAL4 line, in which GAL4 expression is under the control of the 13-kb regulatory element upstream of the first exon of *dve*. Expression pattern of the green fluorescent protein (GFP) driven by the *dve-GAL4[35A]* (*dG35A>GFP*) is nearly identical to that of endogenous Dve protein, although low-level expression in main cells could be detected in the adult stage (Figure 1E–H).

The homeobox gene *paired* (*prd*) is required for accessory gland development and its mutation leads to greatly reduced size of the accessory gland [17]. We compared Dve expression with that of Prd, and found that the onset of their expression was nearly the same (Figure 1I–K). However, Prd expression in main cells is maintained in the adult stage (Figure 1L). In the midgut, Dve is coexpressed with the copper cell determinant Labial in embryonic precursor cells, and the subsequent *dve* repression in the larval copper cells is regulated by Labial and Dve itself [24]. We hypothesized that the temporally regulated *dve* repression in accessory gland main cells is also regulated in a similar manner. Therefore, we examined the effect of *prd* mutation on Dve expression. Interestingly, Dve was derepressed in *prd* mutant main cells, which also had lost their binucleate character (Figure 1M). In contrast, *prd* mutant secondary cells were normally binucleate and Dve expression was maintained in these cells (Figure 1N). Thus, the Prd activity is required for temporally regulated *dve* repression in the main cells and it is dispensable for Dve expression in the secondary cells.

### Dve-A is required for secondary cell development

**Figure 2:**
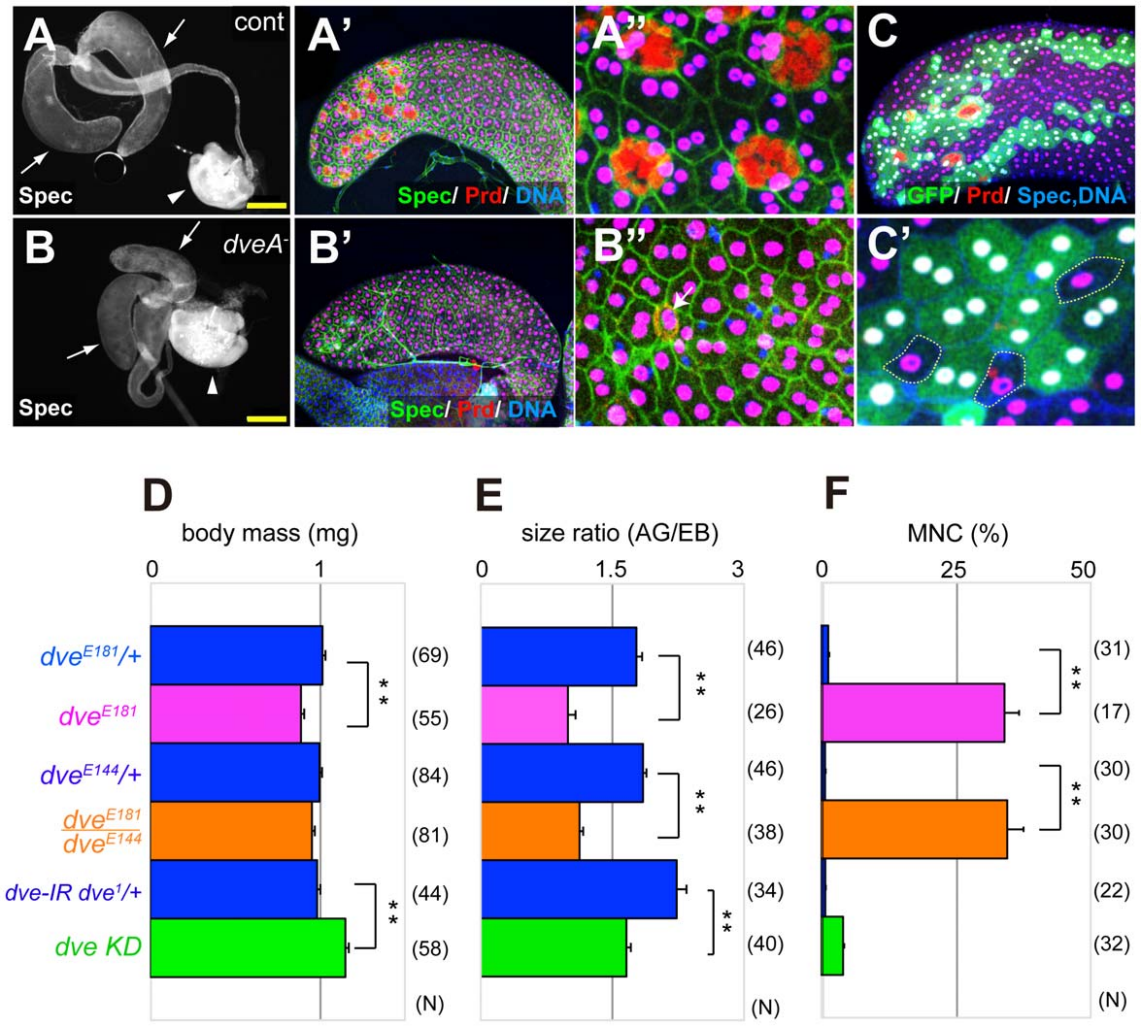
Dve-A is required for secondary cell development. (A and B) Reproductive organs of a control male (A: *dve^E181^/CyOGFP*) and a *dve-A* mutant male (B: *dve^E181^* homozygote). The size of mutant accessory glands (AG, arrows) is small compared with the size of ejaculatory bulb (EB, arrowheads). Scale bar is 200 µm. A control AG has about 20 secondary cells in a half-plane (A′ and A″), and the cytoplasmic vacuolar components are non-specifically stained with rabbit anti-Prd antibody (red in A″). Nuclei (DNA) and cell membrane are labeled with TO-PRO3 (blue) and anti-Spectrin antibody (green), respectively. Binucleate Prd expression is shown in magenta (A′ and A″). A *dve-A* mutant AG has mononucleate main cells that normally express Prd (magenta in B′ and B″). The *dve-A* mutant AG has no mature secondary cells, although the signal for vacuolar components (red) is rarely detected in a very small atrophic cell (arrow in B″). (C and C′) A *dve^1^* mutant mosaic AG of 4–6 days old adult. Mutant clones are marked by the absence of GFP expression (green) and are outlined in C′. No secondary cells are detected within *dve* mutant clones (C), and mononucleate cells are detected only in mutant clones (C′). (D–F) Comparison of body mass (D), size ratio of AG/EB (E), and rate of mononucleate cells (MNC) (F) between males of the indicated genotypes. N indicates the number of individuals tested. Error bars show standard error of means (SEM) (** p<0.001, * P<0.01, t-test). RNAi-mediated *dve* knockdown (*dve* KD: *dG30A>dve-IR dve^1^*) also shows reduced AGs, which have relatively normal binucleation compared with those of *dve-A* mutants.

Two transcripts, type A (4.9 kb) and type B (3.5 kb), have been identified in the *dve* locus. The type-A (*dve-A*) null allele *dve^E181^* has about 4-kb deletion including the first exon for the *dve-A* transcript, while the *dve^E144^* allele has a smaller deletion [22]. Homozygosity for *dve^E181^* is semi-lethal, and escaper adult males have smaller accessory glands (Figure 2A, 2B). Because the body size of *dve^E181^* homozygous males was also small (Figure 2D), we measured the size of ejaculatory bulb as an internal control. The size ratio of cross-section area (accessory gland/ejaculatory bulb, AG/EB) was significantly reduced in males of *dve^E181^* homozygotes and heteroallelic combination (*dve^E181^*/*dve^E144^*) (Figure 2E). Similar phenotype was also observed in RNAi-mediated *dve* knockdown (*dve* KD, *dG30A>dve-IR dve^1^*) (Figure 2E and Figure S1). To perform efficient knockdown, we recombined *UAS-dve-IR* with a loss of function allele, *dve^1^* [24], [25], to generate a recombinant chromosome, *UAS-dve-IR dve^1^*. Notably, these *dve-A* mutant accessory glands completely lacked cells with the appearance of mature secondary cells and some of their main cells were mononucleate (Figure 2B′, 2B″, 2F). Secondary cells can be distinguished by their spherical cell shape and the presence of large vacuoles. The vacuolar components are non-specifically stained with anti-Prd antibody (red in Figure 2A′, 2A″). Interestingly, they were rarely detected in very small atrophic cells of *dve-A* mutants (arrow in Figure 2B″), suggesting that cell fate for the secondary cell could be induced in *dve-A* mutants.

Because *prd* mutant main cells are mononucleate (Figure 1M), we checked whether these *dve-A* mutant phenotypes are due to the absence of Prd activity. Prd expression was unaffected in *dve^E181^* mutants (Figure 2B″), indicating that the Dve activity is crucial for growth of the accessory gland, binucleation of main cells, and secondary cell differentiation even in the presence of Prd proteins. To further confirm the Dve functions in accessory gland development, we performed mosaic analysis using the loss of function allele *dve^1^*. Mutant clones for *dve^1^* showed the same phenotypes as the accessory gland did in a *dve-A* mutant male. Cells with secondary morphology were only detected in GFP-positive wild-type cells but not within *dve^1^* mutant clones (Figure 2C, 2C′).

### Seminal fluid from *dve* mutant males is defective despite the presence of sex peptide

**Figure 3:**
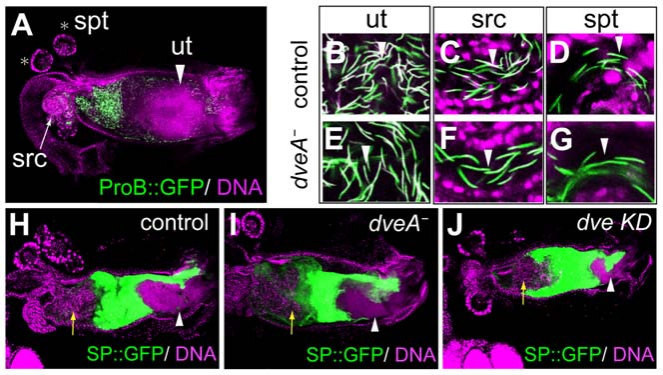
Localization of sperm and SP derived from *dve* mutant males. (A) Female reproductive organs, uterus (ut), seminal receptacle (src), and spermatheca (spt), are visualized with DNA staining (magenta) at 30 min after mating. The Protamine B-GFP fusion proteins (ProB::GFP, green) are localized in the sperm nuclei. (B–G) Localization of control sperms (B–D) and *dve-A* mutant sperms (E–G) in reproductive organs of wild-type females at 30 min after mating. The *dve-A* mutant sperms are correctly localized to the sperm storage organs (src and spt). (H–J) Localization of the sex peptide-GFP fusion proteins (SP::GFP, green) in reproductive organs of wild-type females at 20–30 min after mating. Females mated with control (H), *dve-A* mutant (I), and *dve* KD (J) males are shown. Arrows indicate the sperm mass and arrowheads indicate the posterior mating plugs.

**Figure 4:**
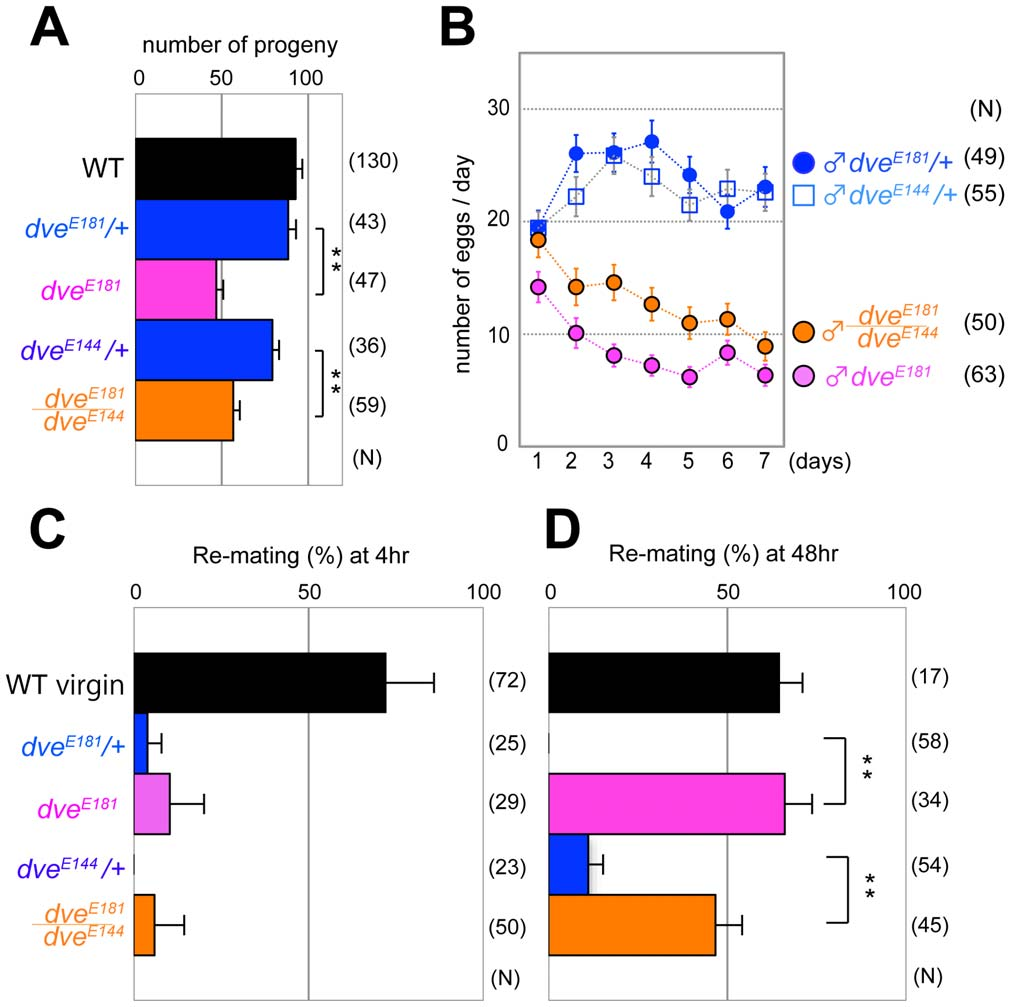
Behaviors of females mated with *dve* mutant males. Postmating response of a wild-type female mated with a male of the indicated genotype. (A) The average number of progeny during 4 days. WT: wild-type male. A female mated with a *dve-A* mutant male (*dve^E181^* or *dve^E181^/dve^E144^*) has reduced number of progeny compared with heterozygous controls. (B) The average number of eggs laid in a day. (C and D) Remating rate with a wild-type male at 4 hr (C) and 48 hr (D) after first mating with the indicated male. Mating rate of WT virgin females is also shown (black bar). Egg laying (B) and remating rate at 48 hr (D) of females mated with *dve* mutant males are comparable to those of virgin female controls. Error bars show SEM (** p<0.001, one-way ANOVA).

As *prd* mutant males who have very small accessory glands are sterile due to a defect in ejaculation [18], [19], [20], we checked the fertility/fecundity of *dve^E181^* mutant males. The *dve-A* mutant sperms had normal motility and they were correctly localized to the female sperm storage organs, seminal receptacle (src) and spermathecae (spt) (Figure 3A–G). However, the average number of progeny from a wild-type female mated with a *dve-A* mutant male was significantly lower than those mated with control males (Figure 4A). Therefore, it is assumed that the low fecundity of *dve-A* mutant males reflects the defects of seminal fluid proteins that are secreted from accessory gland cells. We examined two potential reasons for the low fecundity: (1) failure of egg-laying stimulation, and (2) reduced rate of fertilization as reported in *wasted* mutant sperm [28]. Females mated with *dve-A* mutant males laid substantially fewer eggs than those mated with control males, although the hatching rate was indistinguishable between females mated with *dve-A* mutant or control males (Figure 4B and data not shown). These results strongly suggest that the low fecundity of *dve-A* mutant males is due to the failure of egg-laying stimulation to mated females.

The sex peptide (SP), also known as Acp70A, is a major accessory gland protein that induces changes in female postmating response such as egg laying and receptivity [7], [10]. Females mated with *SP* null mutant males lay fewer eggs, and they show high receptivity to the second males (i.e., remating activity) at least 12 hr after mating. The mating plug protein PEBII, which is secreted from the ejaculatory bulb, contributes to reduced receptivity at earlier period (4 hr after mating) [29]. Thus, we examined remating activity of a female firstly mated with a *dve-A* mutant male and secondly with a wild-type male. The remating activity at 4 hr after first mating was not evident (Figure 4C). Interestingly, females at 48 hr after first mating with *dve-A* mutant males showed high remating activity (Figure 4D). These results raised the possibility that SP expression or its transfer is impaired in *dve-A* mutant males. Thus, the SP expression in main cells was monitored using a GFP fusion protein (SP::GFP), whose expression is under the control of the endogenous *SP* promoter [30]. Unexpectedly, SP::GFP was normally expressed and secreted into the lumen of the *dve-A* mutant accessory glands, and it was normally transferred into the female reproductive tract after mating (Figure 3H–J).

### Secondary cells are essential for male fecundity

**Figure 5:**
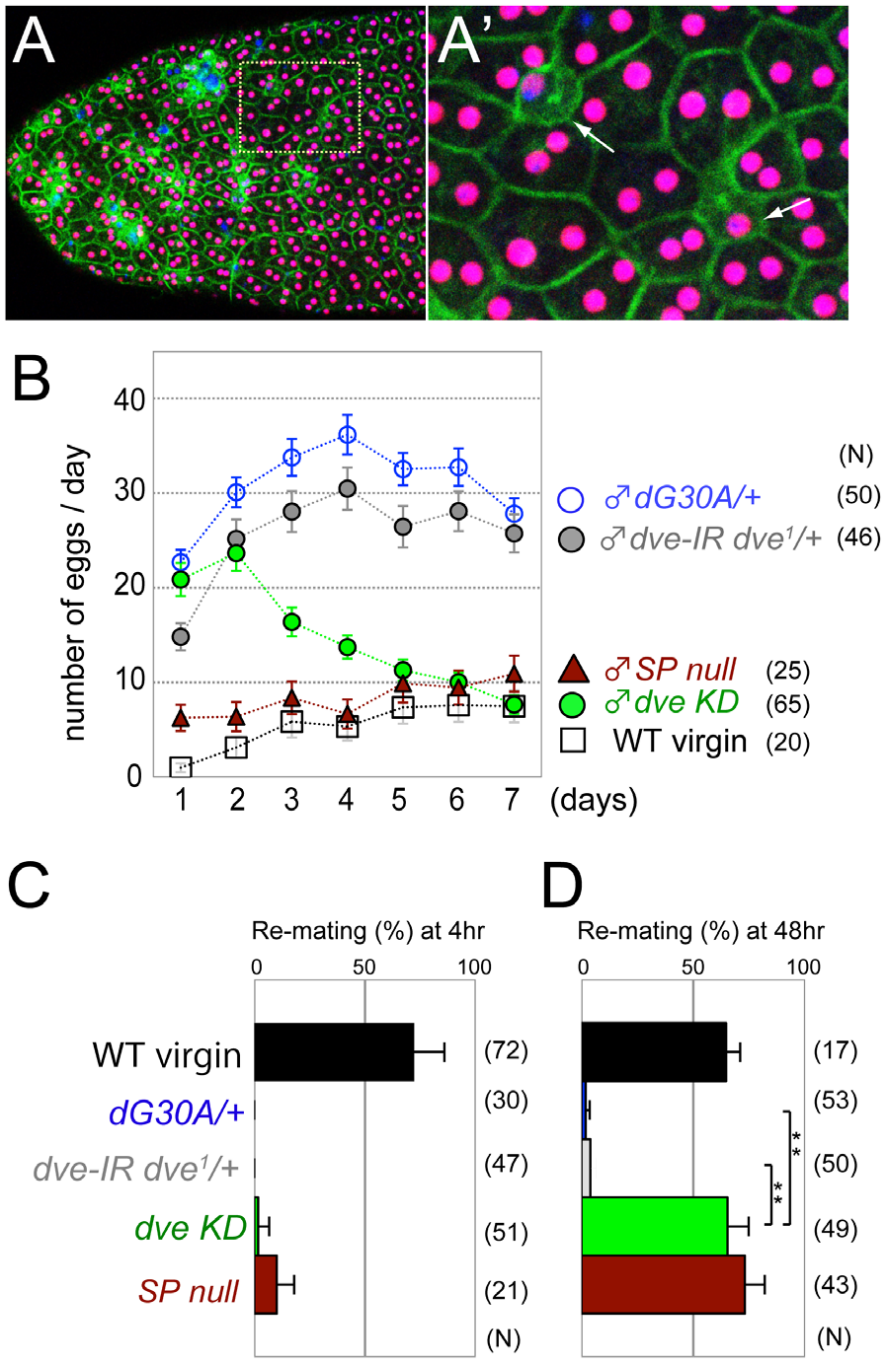
Behaviors of females mated with *dve* KD males. (A) An accessory gland in which *dve* is knocked down (*dve* KD, *dG30A>dve-IR dve^1^*). Nuclei and cell membrane are labeled with anti-Prd (magenta) and anti-Spectrin (green) antibodies, respectively. The boxed region is magnified in (A′) and arrows indicate small atrophic secondary cells. (B–D) Postmating response of a wild-type female mated with a male of the indicated genotype. (B) The average number of eggs laid in a day. (C and D) Remating rate with a wild-type male at 4 hr (C) and 48 hr (D) after first mating with the indicated male. Mating rate of WT virgin females is also shown (black bar). Egg laying (B) and remating rate at 48 hr (D) of females mated with *dve* KD males are comparable to those of virgin female controls or females mated with *SP* null mutant males (*SP^0^/Δ130*). Error bars show SEM (** p<0.001, one-way ANOVA).

To examine whether the low fecundity of *dve* mutant males is due to the mononucleate state of SP-expressing main cells, we checked the postmating response of females after they had mated with *dve* KD males. In *dve* KD males, binucleation of main cells was relatively normal in contrast to mononucleate main cells seen in *dve-A* mutants (Figure 2F and Figure 5A). This does not mean that *dve* KD main cells are functionally normal, however, secondary cell differentiation was severely inhibited and small atrophic cells were frequently observed (Figure 5A′). Females mated with the *dve* KD or *dve-A* mutant males showed the same phenotypes in egg laying and remating activity (Figure 5B–D), suggesting the importance of mature secondary cells for male fecundity.

Although little is known about physiological significance of secondary cells, *Abd-B* mutant males also lack mature secondary cells and show low fecundity similar to that of *dve-A* mutants (personal communication from M. F. Wolfner and F. Karch). These results further support the importance of secondary cells and prompted us to examine the relationship between Dve and Abd-B during secondary cell development. Expression patterns of Dve and Abd-B are nearly complementary until 24 hr APF and change to the same pattern from 48 hr APF to the adult stage (Figure S2A–H). Abd-B expression after the late pupal stage appears to be dependent on the Dve activity, because it was greatly reduced in *dve* mutant clones and also in adjacent wild-type cells (Figure S2E). Thus, it seems likely that the low fecundity in *dve* mutant males reflects the absence of Abd-B expression with loss of mature secondary cells. Taken together, these results strongly suggest that unknown factors secreted from secondary cells are essential for male fecundity.

### The common region of Dve isoforms is required for survival of secondary cells

**Figure 6:**
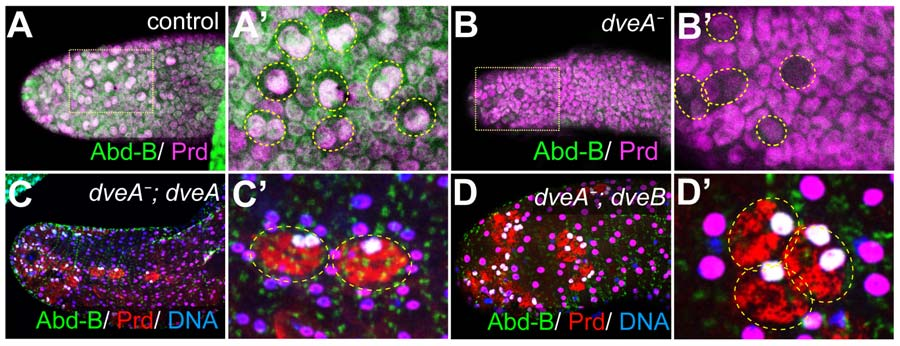
Secondary cell precursors are detectable in *dve* mutants. (A and B) Accessory glands of a control male (A: *dve^E181^/CyOGFP*) and a *dve-A* mutant male (B: *dve^E181^/dve^E144^*) at 72 hr APF. Nuclei are labeled in magenta with anti-Prd antibody. The control secondary cell precursors (outlined in A′) strongly express Abd-B (green). A *dve-A* mutant accessory gland has a few secondary cell precursors in which Prd expression is greatly reduced (outlined in B′). (C and D) Accessory glands of 4-days old adults are labeled with anti-Prd (red) and anti-Abd-B (green) antibodies. (C) Loss of secondary cells in *dve-A* mutants is rescued by expressing *dve-A* (*w; dve^E181^/dve^E144^; dG30A/UAS-dveA-9B2*). (D) Loss of secondary cells in *dve-A* mutants is also rescued by expressing *dve-B* (*w; dve^E181^/dve^E144^; dG30A/UAS-dveB-Y4N*). The rescued secondary cells are outlined. They express Abd-B (white in C′ and D′) and have vacuole components that are non-specifically stained with anti-Prd antibody (red in C′ and D′).

In *dve-A* mutant or *dve* KD accessory glands, we could detect very small atrophic cells (Figure 2B″ and Figure 5A′). These observations suggest a mechanism that Dve is required for cell survival rather than cell-fate determination of secondary cells. Consistent with this notion, we could detect a few secondary cell precursors in *dve-A* mutants during pupal development. These mutant cells had a characteristic feature of secondary cells in their nuclear arrangement and also showed reduced Prd expression (Figure 6A, 6B). Because Prd expression in main cells was unaffected in *dve* mutants (Figure 2B′, 2C and Figure S2E), these cells appear to be precursors of atrophic secondary cells and they were detected only in the distal region (Figure 6B). It seems likely that most precursor cells rapidly die in *dve-A* mutants, because it was quite difficult to detect these precursor cells even in earlier stages.

Loss of secondary cells in *dve-A* mutants was rescued by *dve-A* expression (Figure 6C). Interestingly, expression of *dve-B* also rescued loss of secondary cells in the *dve-A* mutant background (Figure 6D). These results imply that the common region of two isoforms including a compass domain (CMP) and two homeodomains (HD-N and HD-C) is responsible for survival of secondary cells.

## Discussion

**Figure 7:**
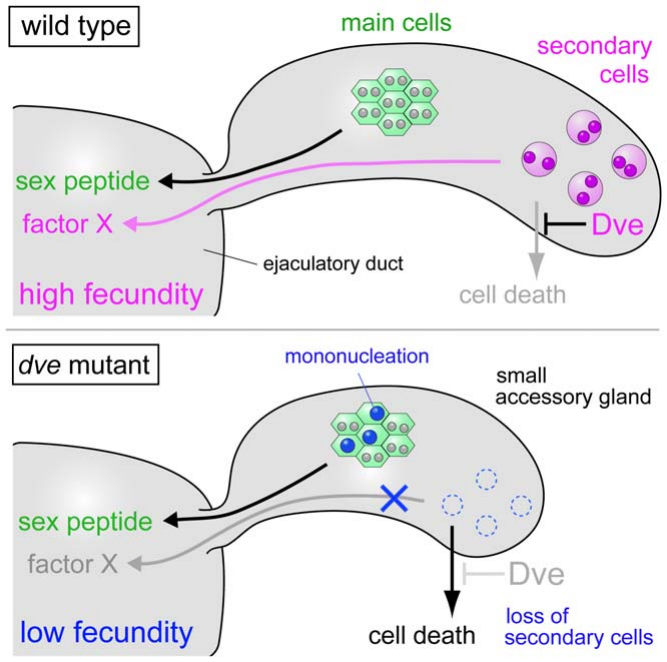
Schematic diagram of Dve functions during accessory gland development. Dve is strongly expressed in adult secondary cells (magenta) and undetectable in adult main cells (green). The main cells secrete sex peptide that is essential for long-term postmating response to increase progeny production. The Dve activity is required for proper development of these cells: survival of the secondary cells and binucleation of the main cells. An unknown factor X secreted from the secondary cells is essential for increasing progeny production, i.e., high fecundity.

Mutant males for *dve-A* showed low fecundity together with loss of secondary cells and reduced size of accessory glands. It has been reported that greatly reduced size of accessory glands results in sterility, and also suggested that there is a minimum size to maintain fertility [31]. If a male is selected for larger size of accessory glands with 16 generations, the selected males have good fecundity. However, they have only about 1.4-fold larger size compared to the control males, suggesting that there is also a maximal size of accessory gland not to waste energy [31]. Thus, the size of accessory glands should be controlled in an appropriate range and binucleation seems to be the best strategy to provide highly plastic change of the size [32]. Although reduced size of *dve-A* mutant accessory glands may have some effects on fecundity, *dve* KD males had similar size of accessory glands to the *dve-A* heterozygous controls and *dve* KD males showed low fecundity with loss of secondary cells (Figure 2E and Figure 5). Thus, it is most likely that the low fecundity in *dve-A* mutant males is due to the absence of mature secondary cells (Figure 7). This is consistent with independent findings that Abd-B is required for maturation of secondary cells and for maintaining female postmating response (personal communication from M. F. Wolfner and F. Karch). Although we cannot exclude the possibility that some defects in *dve* mutant main cells affect the fecundity, SP was normally expressed and transferred into the female reproductive tract (Figure 3H–J). Thus, the following mechanisms should be considered for SP activation to induce long-term postmating response; (1) secondary cell products cooperate in parallel with SP-mediated signaling; (2) secondary cell products enhance SP binding to its receptor; (3) secondary cell products stabilize SP binding to sperm; and (4) secondary cell products are involved in modification and/or stabilization of SP secreted from main cells. Interestingly, egg laying of females mated with *dve* mutant or *dve* KD males was gradually reduced over time (Figure 4B, 5B), suggesting that the last two interpretations, stabilization of SP by secondary cell products, are more plausible. Identification of unknown factors secreted from secondary cells will provide new insights into a mechanism that is crucial for activation of seminal fluid functions.

It seems likely that Dve functions are crucial to inhibit cell death of secondary cells, and an intriguing possibility is that inactivation of the Dve activity is closely linked to the regulated cell death to adjust the number of secondary cells. Dve and the special AT-rich sequence binding proteins (SATBs) belong to the *cut* superclass of homeobox genes and have an evolutionarily conserved CMP [33]. It is reported that SATB1 is cleaved and inactivated by Caspase 6 in response to the apoptotic signaling pathway [34]. Expression of the BCL2 gene, which is a key regulator to inhibit apoptosis, is finely tuned by a variety of stimuli and activated through SATB1-mediated chromatin looping. Thus, SATB1 is required for cell survival through inhibition of programmed cell death [35], [36]. The functional similarity between Dve and SATB1 for inhibition of cell death raises a possibility that an evolutionarily conserved CMP plays important roles to inhibit cell death. The CMP of SATB1 is characterized as a PDZ-like domain (amino acids 90 to 204) involved in protein-protein interaction, and the Caspase 6-dependent cleavage of SATB1 at amino acid position 254 disrupts the dimerization of SATB1. Further characterization of CMP-interacting proteins will clarify the underlying mechanism and provide new insights into a regulatory mechanism of fecundity/fertility.

## Materials and Methods

### Fly stocks

Flies were reared on standard yeast-glucose-cornmeal-agar medium at 25°C. Oregon-R flies were used as wild-type controls. *dve^E181^* and *dve^E144^* are *dve-A*-specific mutant alleles that remove the first exon [22]. *dve^1^* is a severe loss of function allele that has no *dve-A* and a very weak *dve-B* activity in the larval midgut [22], [24]. *dve^L186^* is a null allele that completely removes the gene [37]. To knockdown the *dve* activity in a spatio-temporal manner, we used *UAS-dve-IR* (v109538, Vienna Drosophila RNAi Center), which can induce RNA interference by expressing the inverted repeat (IR) sequence of exon 3. To perform efficient knockdown, a recombinant line, *UAS-dve-IR dve^1^*, was used for experiments. *SP* null mutant males were produced by crossing *SP^0^/TM3 Sb* males with *Δ130/TM3 Sb* females. The resulting *SP^0^/Δ130* (*SP* null) males produce no SP [10]. The following GFP marker strains were used: protamineB::GFP for sperm nuclei (Drosophila Genetic Resource Center, DGRC) [38] and SP::GFP for expression of sex peptide [30].

The following GAL4/UAS lines were used: *btl-GAL4*, *UAS-flp*, *UAS-GFP.nls* (Bloomington), *UAS-GFP-N-lacZ* (DGRC), *UAS-dveA-9B2*, and *UAS-Flag-dveB-Y4N* (on the third chromosome) [22], [24].

### Establishment of *dve-GAL4* lines

The *lacZ* sequence of pCaSpeR-AUG-β-gal vector [39] was replaced with the *GAL4* sequence from pGaTB [40]. This pCaSpeR-GAL4 vector has unique restriction enzyme sites (*Kpn*I, *Eco*RV, and *Bam*HI) upstream of the *GAL4* open reading frame. The 5.8-kb fragment, which includes the upstream promoter region and the first exon (to +557) of the *dve* gene, was inserted into the *Kpn*I-*Bam*HI sites of pCaSpeR-GAL4 to generate pCaSpeR-5.8EΔE-GAL4. The 5.4-kb fragment, which includes further upstream sequences, was inserted into the *Kpn*I-*Eco*RI sites of pCaSpeR-5.8EΔE-GAL4 to generate pCaSpeR-5.4-5.8E-GAL4. The 1.6-kb *Eco*RI fragment was inserted into the junctional *Eco*RI site between 5.4-kb and 5.8-kb fragments of pCaSpeR-5.4-5.8E-GAL4 to generate the *dve-13kb-GAL4*. This construct expresses *GAL4* under the control of upstream 13-kb elements of the *dve-A* promoter, and transgenic flies were established using P-element mediated germ line transformation under standard procedures. Two established lines inserted on the third chromosome, *dve-GAL4[30A]* and *dve-GAL4[35A]*, were used in this study.

### Immunohistochemistry

Larvae, pupae, and adults were dissected in phosphate-buffered saline (PBS), fixed with 4% formaldehyde/PBS-0.3% Triton X-100 for 20 min, and washed several times with PBS-0.3% Triton X-100. The following primary antibodies were used: rabbit anti-Dve (1∶1000) [24], rabbit anti-Prd (1∶100, Asian Distribution Center for Segmentation Antibodies) [41], mouse anti-Abd-B (1∶10) (Developmental Studies Hybridoma Bank, DSHB), mouse anti-α-Spectrin (3A9) (1∶50, DSHB), and mouse anti-β-galactosidase (1∶200, Promega). FITC-, Cy3- or Cy5-conjugated secondary antibodies (Jackson Immunoresearch) were used for detection. TO-PRO3 (Invitrogen) was used to detect DNA. Confocal images were obtained with an OLYMPUS FV300. Quantification of a cross-section area was performed using a measurement module (BZ-H1M, KEYENCE).

### Mosaic analyses

Mutant mosaic clones were induced with the use of FRT- and FLP-mediated recombination system [42] as following genotypes: *w; FRT42D ubiGFP/FRT42D dve^1^; btl-GAL4/UAS-flp; w; FRT42D ubiGFP M(2)53^1^/FRT42D dve^L186^; btl-GAL4/UAS-flp; y w hsflp; ubi-GFP FRT40A/prd^4^ FRT40A* with a heat shock pulse at 38°C-15 min. Mosaic clones expressing *dve-IR* (*y w hs-flp*; *Ay-GAL4 UAS-GFP.S65T*/*UAS-dve IR dve^1^*) were induced with the Ay-GAL4 system [43] with a heat shock pulse at 38°C-20 min.

### Behavior assays

All flies were cultured at 25°C under 12 hr light/dark cycles. Males and females were collected at eclosion and aged separately for 4–6 days in groups of 10–30 flies per vial. At circadian times 0 to 3 (CT0, subjective dawn), a pair of male and female flies was placed on a food medium with an observation chamber (30-mm diameter×4-mm depth) for 1 hr. A mated female was mildly aspirated into a vial and used for the following assays.

#### Progeny assay

Male fertility/fecundity was measured as the number of viable progeny per mated wild-type female during 4 days after mating. A mated female was transferred twice to a fresh vial and allowed to lay eggs for 2 days. The number of adult progeny from each vial was counted.

#### Egg-laying/hatching assay

A mated female was transferred to a fresh vial every 24 hr for 7 days, and eggs laid per day were counted. Hatching rate was determined by counting the number of unhatched eggs during next 24 hr.

#### Remating assay

At 4 hr and 48 hr after first mating, mated females were individually paired with one new virgin male (4–6 days old) for 1 hr. Several independent sets of remating assay were performed and the cumulative percentage of remated females was calculated.

### Statistical analyses

The significance of differences between the control and test progenies was analyzed with t-tests or one-way ANOVA using Kaleidagraph software version 3.6 (HULINKS). The levels of significance are indicated by asterisks: *P<0.01, **P<0.001.

## Supporting Information

Figure S1 **RNAi-mediated** ***dve*** **knockdown in the accessory gland.** RNAi-mediated *dve* knockdown (KD) greatly reduced Dve protein level (magenta) in the proximal (A) and the distal regions (B) at 72 hr APF. Cells inducing *dve* RNAi are marked by GFP expression (green: *y w hs-flp*; *Ay-GAL4 UAS-GFP.S65T*/*UAS-dve IR dve^1^*), and nuclei (DNA) are labeled with TO-PRO3 (blue).
(TIF)

Figure S2 **Dve-dependent Abd-B expression during late pupal development.** (A–D) Expression of Dve (A–D, magenta) and Abd-B (A′–D′) proteins in a male genital disc (A), accessory gland (AG) primordia at 24 hr APF (B), 48 hr APF (C), and AG of 1-day old adult (D). Abd-B is expressed in accessory gland precursors (A′) marked by *btl* expression (light blue in A), and transiently repressed at 24 hr APF (B′). After 48 hr APF, expression patterns of Dve and Abd-B are nearly identical. MC: main cells (arrowheads), SC: secondary cells (arrows). (E) Abd-B expression (magenta in E′) is greatly reduced in *dve^L186^* null mutant clones at 72 hr APF. Mutant clones are marked by the absence of GFP expression (green) and are outlined in E″. Some wild-type cells also show reduced Abd-B expression in a cell-non-autonomous manner. ED: ejaculatory duct.
(TIF)
