# Supplementary figures and images for: The Homeodomain Protein Defective Proventriculus Is Essential for Male Accessory Gland Development to Enhance Fecundity in Drosophila
Source: PLoS One. 2012 Mar 12;7(3):e32302. doi: 10.1371/journal.pone.0032302 (PMC3299662; doi:10.1371/journal.pone.0032302)

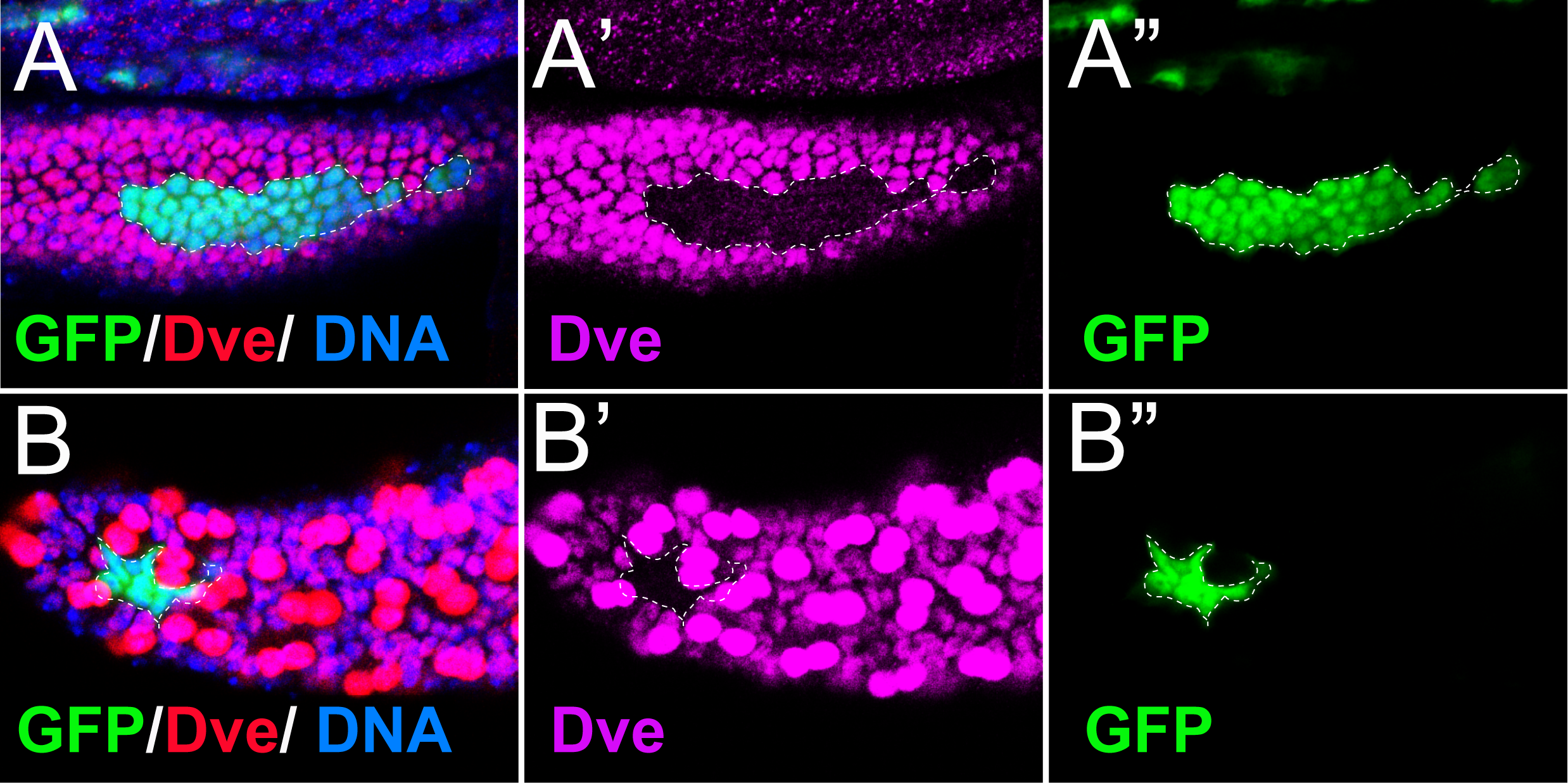

Supplement: Figure S1 — RNAi-mediated dve knockdown in the accessory gland. RNAi-mediated dve knockdown (KD) greatly reduced Dve protein level (magenta) in the proximal (A) and the distal regions (B) at 72 hr APF. Cells inducing dve RNAi are marked by GFP expression (green: y w hs-flp; Ay-GAL4 UAS-GFP.S65T/UAS-dve IR dve1), and nuclei (DNA) are labeled with TO-PRO3 (blue). (TIF) [file pone.0032302.s001.tif]

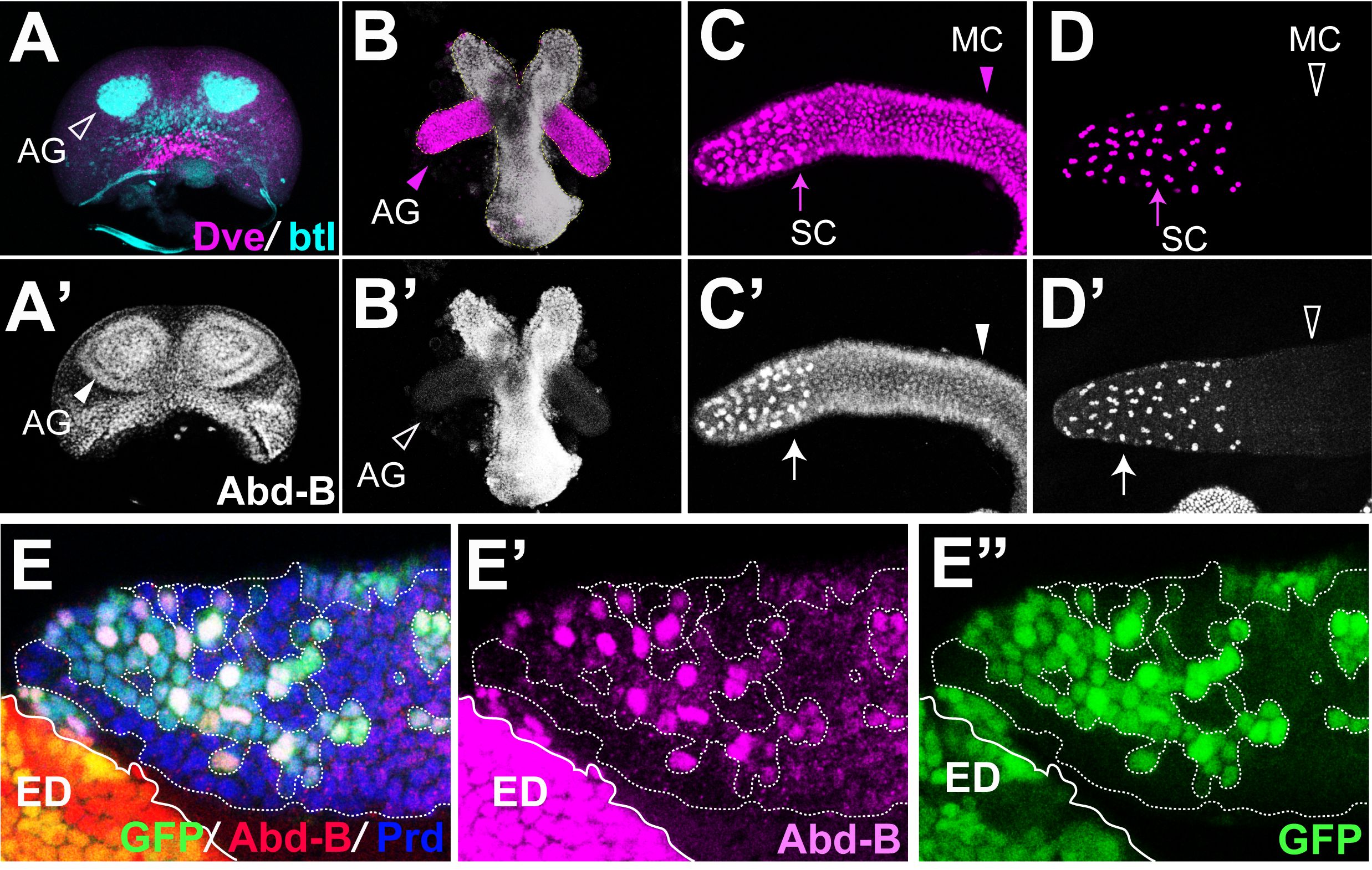

Supplement: Figure S2 — Dve-dependent Abd-B expression during late pupal development. (A–D) Expression of Dve (A–D, magenta) and Abd-B (A′–D′) proteins in a male genital disc (A), accessory gland (AG) primordia at 24 hr APF (B), 48 hr APF (C), and AG of 1-day old adult (D). Abd-B is expressed in accessory gland precursors (A′) marked by btl expression (light blue in A), and transiently repressed at 24 hr APF (B′). After 48 hr APF, expression patterns of Dve and Abd-B are nearly identical. MC: main cells (arrowheads), SC: secondary cells (arrows). (E) Abd-B expression (magenta in E′) is greatly reduced in dveL186 null mutant clones at 72 hr APF. Mutant clones are marked by the absence of GFP expression (green) and are outlined in E″. Some wild-type cells also show reduced Abd-B expression in a cell-non-autonomous manner. ED: ejaculatory duct. (TIF) [file pone.0032302.s002.tif]
